# A novel methylated analogue of *L*-Mimosine exerts its therapeutic potency through ROS production and ceramide-induced apoptosis in malignant melanoma

**DOI:** 10.1007/s10637-021-01087-5

**Published:** 2021-02-23

**Authors:** Sotiris Kyriakou, William Cheung, Theodora Mantso, Melina Mitsiogianni, Ioannis Anestopoulos, Stephany Veuger, Dimitris T. Trafalis, Rodrigo Franco, Aglaia Pappa, David Tetard, Mihalis I. Panayiotidis

**Affiliations:** 1 Department of Applied Sciences, Northumbria University, Newcastle Upon Tyne, UK; 2 Department of Cancer Genetics, Therapeutics & Ultrastructural Pathology, The Cyprus Institute of Neurology & Genetics, Nicosia, Cyprus; 3 The Cyprus School of Molecular Medicine, The Cyprus Institute of Neurology & Genetics, Nicosia, Cyprus; 4 Department of Pharmacology, Medical School, National & Kapodistrian University of Athens, Athens, Greece; 5 Redox Biology Centre, University of Nebraska, Lincoln, USA; 6 School of Veterinary Medicine & Biomedical Sciences, University of Nebraska, Lincoln, USA; 7 Department of Molecular Biology & Genetics, Democritus University of Thrace, Alexandroupolis, Greece

**Keywords:** Metal chelators, Melanoma, Oxidative stress, Glutathione, Ceramide, Apoptosis

## Abstract

Melanoma is an aggressive and highly metastatic type of skin cancer where the design of new therapies is of utmost importance for the clinical management of the disease. Thus, we have aimed to investigate the mode of action by which a novel methylated analogue of *L-*Mimosine (e.g., *L-*SK-4) exerts its therapeutic potency in an in vitro model of malignant melanoma. Cytotoxicity was assessed by the Alamar Blue assay, oxidative stress by commercially available kits, ROS generation, caspase 3/7 activation and mitochondrial membrane depolarisation by flow cytometry, expression of apoptosis-related proteins by western immunoblotting and profiling of lipid biosynthesis by a metabolomic approach. Overall, higher levels of ROS, sphingolipids and apoptosis were induced by *L-*SK-4 suggesting that the compound’s therapeutic potency is mediated through elevated ROS levels which promote the upregulation of sphingolipid (ceramide) biosynthesis thus leading to the activation of both extrinsic and intrinsic apoptosis, in an experimental model of malignant melanoma.

**Supplementary Information:**

The online version contains supplementary material available at 10.1007/s10637-021-01087-5.

## Introduction

Reactive oxygen species (ROS) are generated as by-products of oxygen consumption in mitochondria and/or from other exogenous sources such as pollutants, tobacco, xenobiotics, radiation, etc. [1, 2].

The beneficial role of ROS has been demonstrated multiple times in the literature like in the case of immune response induction [3]. More specifically, activation of surveillance receptors leads to elevation of ROS that is essential for the release of pro-inflammatory cytokines [interleukin 1β (IL-1β), tumor necrosis factor α (TNFα) and interferon β (IFN-β)] which are required for the activation and regulation of an appropriate immune response. Consequently, low levels of ROS can lead to immunosuppression by preventing activation of an immune response [4–6]. Moreover, ROS can influence cell proliferation through regulation of cell cycle progression by acting as second messengers capable of mediating the phosphorylation and ubiquitination of CDKs via activation of the EGF-receptor [7–9].

On the other hand, increased levels of ROS can cause undesirable effects. For instance, they contribute to carcinogenesis through lipid peroxidation, protein carbonylation and DNA/RNA oxidative damage all of which can lead to genetic instability and tumorigenesis [9–13]. However, excessive accumulation of ROS, in cancer cells, can lead to anti-tumorigenic effects by means of rendering them vulnerable to the activation of apoptotic cell death. Thus, cancer therapeutic strategies are designed with the scope to induce ROS generation and consequently trigger an apoptotic response [14, 15]. To this end, it has been previously demonstrated that many chemotherapeutic drugs have the capacity to inhibit the intracellular antioxidant response and thus cause accumulation of ROS which allows for drug-induced cytotoxicity [15]. Nevertheless, the main disadvantage of such approach(es) is the lack of specificity as chemotherapeutic agents cannot target selectively cancer cells [13].

The anti-cancer activity of hydroxypyridone-based (HOPO) metal chelators has been documented, in the past, by others [16, 17] as well as by our research group [18]. More specifically, we have previously shown that treatment of malignant melanoma (A375) cells with a novel *N*-substituted 3,4-HOPO compound (*L-*SK-4) can induce the activation of both intrinsic and extrinsic apoptotic cascades as well as perturbations in cell cycle and ROS homeostasis [18]. Interestingly, an immortalized, non-tumorigenic keratinocyte cell line was shown to be more resistant to the action of *L-*SK-4; thus, proving the compound’s specificity against its potential target. Consequently, we have employed an in vitro model of malignant melanoma consisting of non-metastatic (A375 and B16F-10) as well as metastatic (VMM1 and Hs294T) melanoma cell lines in addition to non-melanoma epidermoid carcinoma (A431) and non-malignant immortalized melanocyte neighbouring keratinocyte (HaCaT) cells. The aim of this study was to investigate the mode of action by which *L-*SK-4 activates the apoptotic response in order to delineate the underlying mechanisms by which it exerts its previously described therapeutic potency [18].

## Materials and methods

### Chemicals

SK-1, SK-2, SK-3, *D*-SK-4, *L*-SK-4 and SK-5 were previously synthesized, purified and characterized [18]. GSH, resazurin sodium salt and Myriocin were purchased from Sigma-Aldrich (St. Louis, MO, USA). All media and cell culture material were obtained from LabTech International Ltd. (East Sussex, UK). Bovine Serum Albumin (BSA) was obtained from Biosera (Boussens, France). Protease and phosphatase inhibitor cocktails were obtained from Roche (Base, Switzerland). Polyvinylidene difluoride (PVDF) membranes (0.45 and 0.2 μm) were purchased from Millipore (Bedford, MA, USA). All solvents were of UHPLC optima grade or better.

### Cell lines

A375 and A431 cells were purchased from Sigma-Aldrich (St. Louis, MO, USA). In addition, VMM1, Hs294T and B16F10 cells were obtained from LGC Standards (Middlesex, UK). All cell lines were maintained in a humidified atmosphere at 37 °C, 5% CO_2_ and according to the provider’s recommended culture conditions. All media and reagents were purchased from Labtech (East Sussex, UK). All plasticware were obtained from Corning (Corning, NY, USA).

### Determination of cell viability

Briefly, A375 cells were seeded in 100 μL/well into 96-well plates and incubated overnight. On the following day, cells were exposed to either GSH (1.5–3.0 mM) or Myriocin (0–50 μM) with or without *L-*SK-4 (100 μM) over different incubation periods while control cells were incubated with complete medium only. The Alamar-blue assay was utilized as previously described [18].

### Determination of ROS

Cells were seeded in 60 mm dishes and then exposed to *L-*SK-4 (100 μM) with or without pre-treatment with either GSH (1.5 mM) or Myriocin (50 nM). Then, they were harvested and washed twice with PBS after which, a single cell suspension of 10^6^ cells/mL was prepared. Dihydrorhodamine 123 (DHR 123; 10 μM) was added in the suspension and incubated for 5 mins at 37 °C followed by addition of DAPI (1 μM), in each sample, and further incubation for 5 mins. Data acquisition and analysis of 10,000 events, for each sample, was performed using a FACS Canto II flow cytometer (BD Biosciences, San Jose, CA, USA). DAPI-positive cells were excluded from further analyses.

### Determination of apoptosis

The CellEvent Caspase 3/7 Green flow cytometry assay kit was utilized for the detection of apoptosis according to the manufacturer’s instructions. Briefly, cells were seeded and allowed to adhere overnight in 60 mm dishes and then exposed to *L-*SK-4 (100 μM) with or without pre-treatment with either GSH (1.5 mM) or Myriocin (50 nM). Next, cells were harvested and washed twice with PBS after which, a single cell suspension of 10^6^ cells/mL was prepared. Then, 0.5 μL of CellEvent Caspase 3/7 Green detection reagent was added into 0.5 mL of each cell suspension and samples were incubated at 37 °C for 30 mins followed by addition of DAPI (1 μM), in each sample, and further incubation for 5 mins. Data acquisition and analysis of 20,000 events, for each sample, was performed using a FACS Canto II flow cytometer (BD Biosciences, San Jose, CA, USA).

### Determination of mitochondria membrane depolarization

The JC-1 staining solution was used according to the manufacturer’s instructions. Following treatment with *L-*SK-4 (100 μM) with or without pre-treatment with myriocin (50 nM), cells were harvested and washed twice in PBS. Then, 0.3 μL of JC-1 (0.1 mg/mL) was added into 0.3 mL of each cell suspension, in PBS, and samples were incubated at 37 °C for 30 mins. All cell suspensions were centrifuged at 1000 rpm for 5 mins and pellets were re-suspended in fresh PBS. Data acquisition and analysis of 10,000 events, for each sample, was performed using a FACS Canto II flow cytometer (BD Biosciences, San Jose, CA, USA).

### Preparation of cell lysates and protein determination

A375 cells were plated in 100 mm dishes and cultured overnight at 37 °C. Next day, cells were treated with *L-*SK-4 (100 μM) with or without pre-treatment with either GSH (1.5 mM) or myriocin (50 nM) for 24, 48 and 72 h. Cell lysates were prepared and obtained as previously described [18]. Protein content was determined by utilizing the BCA protein assay kit (Thermo Scientific, Waltham, MA, USA), according to the manufacturer’s protocols. Protein extracts were stored at −20 °C until usage.

### Western immunoblotting

Standard conditions were used as previously described [18]. All antibodies (e.g., anti-Caspases-8 and -9, anti-Apaf-1, anti-BID, anti-FADD, anti-FAS, anti-BAX, anti-BAK and anti-Tubulin) were purchased from Cell Signaling Technology (Danvers, MA, USA) and utilized according to the manufacturer’s protocol.

### Determination of lipid peroxidation content

A375 cells were plated in 100 mm dishes, cultured overnight and next day were treated with *L-*SK-4 (100 μM). After trypsinization, pellets were collected, re-suspended and sonicated before the TBARS Assay kit (Cambridge Bioscience Ltd., Cambridge, UK) was utilized for the determination of malondialdehyde (MDA) content according to the manufacture’s protocol.

### Determination of protein carbonyl content

A375 cells were plated in 100 mm dishes, cultured overnight and next day were treated with *L-*SK-4 (100 μM). After trypsinization, pellets were collected, resuspended and sonicated before the Protein Carbonyl Colorimetric Assay Kit (Cambridge Bioscience Ltd., Cambridge, UK) was utilized for the determination of protein carbonyl content according to the manufacture’s protocol.

### Determination of oxidative DNA damage content

A375 cells were plated in 100 mm dishes, cultured overnight and next day were treated with *L-*SK-4 (100 μM). After trypsinization, pellets were collected and the dsDNA content was extracted using the PureLinK™ Genomic DNA Mini Kit (Invitrogen, Carlsbad, CA, USA) and then converted to ssDNA according to the manufacture’s protocol. The DNA/RNA Oxidative Damage (High Sensitivity) ELISA Kit (Cambridge Bioscience Ltd., Cambridge, UK) was utilized for the determination of 8-oxo-2-deoxy guanosine (8-OHdG) content according to the manufacture’s protocol.

### Lipidomic extraction protocol

A375 cells were plated in 100 mm dishes and cultured overnight at 37 °C. Next day, cells were treated with *L-*SK-4 (100 μM) and then trypsinised and collected by centrifugation at 2000 rpm for 3 mins at 4 °C. Approximately 3 × 10^6^ cells were washed with ice-cold PBS three times prior to extraction. Then, the cell pellet was re suspended in 300 μL of the extraction buffer (dichloromethane/methanol (3:1 *v*/v) chilled to 4 °C) and cell lysis was induced by snap freezing the samples in liquid nitrogen for 1 min and thaw over ice. This was repeated 5 times to ensure complete cell lysis. Then, cell suspensions were sonicated for 15 mins and ultra-centrifuged at 15,000 rpm for 15 mins. The entire supernatant was transferred to 1.5 mL Eppendorf and allowed to evaporate, at RT, overnight under a fume hood. The dried down extracts were reconstituted in 300 μL of isopropyl alcohol/ACN/water (2:1:1), sonicated for 15 mins and ultra-centrifuged at 15,000 rpm for an additional 15 mins before transferring 100 μL to 1.5 mL autosampler vial, caped and then subjected to lipidomic characterization.

### Lipidomic sample analysis

*LC parameters:* Chemical analysis was performed on a Thermo Scientific Orbritap classic mass spectrometer hyphenated to a Dionex 3000 UHPLC with the autosampler tray set to 4 °C. The separation was performed on a Waters C18 CSH analytical column, 2 × 100 mm with a 1.7 μm particle size. The column was maintained at 55 °C with a flow rate of 400 μL/min. A binary buffer system was used for the chromatographic separation. Buffer A was 60/40 (*v*/v ACN/MillQ water) and Buffer B (90/10 v/v isopropyl alcohol and ACN) with 10 mM ammonium formate and 0.1% formic acid.

*LC profile:* Starting condition: 00.00 min 45% (B) → 11.00 min 65% (B) → 20.00 min 99% (B) → 24.00 min 99% (B) → 24.25 min 45% (B) → 28.50 min 45% (B).

*Mass spectrometer:* The HESI source condition was as follows: Sheath gas flow: 50, Aux Gas flow: 13, Sweep gas flow: 3. Spray voltage was set 3.5 kV, Capillary temperature was set to 275. S-lens RF level was set to 50 and the temperature of the HESI was set to 425 °C.

*Mass spectral acquisition parameter:* Scan range was set from 300 to 2000 m/z at mass resolution of 140 K with a scan rate of 1.6 scans/s with an automatic gain control of 1 × 10^6^ with a maximum injection time of 100 ms. MS1 profiling in positive polarity mode.

*Peak table generations:* Compound discoverer V2, the alignment window was set to 0.25 mins with mass tolerance of 5 ppm with (M-H)^+^ adducts only. Quality control and extraction blanks were imbedded into the analysis for stability assessment and background subtractions.

### Statistical analysis

Data were expressed as mean values ± standard deviation (SD) and comparisons were made between control and treated groups. Statistical analyses were performed by one-way ANOVA with Tukey’s test for multiple comparisons, by using the SPSS v.22 software, and statistical significance was set at *p* < 0.05, *p* < 0.01 and *p* < 0.001. For the lipidomic analyses, all multivariate data analyses were performed using the metaboanalyst v3 web-based version (https://academic.oup.com/bioinformatics/article/34/24/4313/5046255).

## Results

### The capacity of HOPOs to induce ROS production

**Fig. 1:**
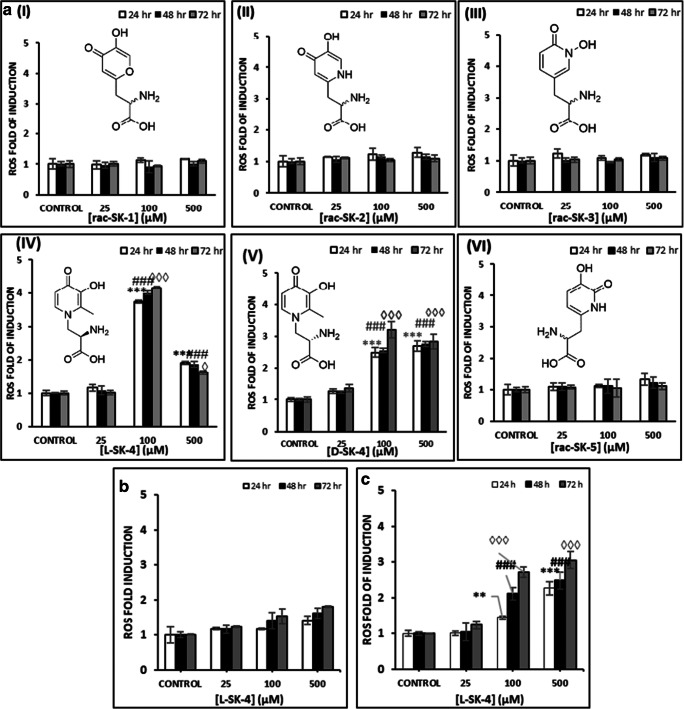
Induction of ROS generation by various hydroxypyridinone compounds in melanoma (A375), non-melanoma epidermoid carcinoma (A431) and non-tumorigenic keratinocyte (HaCaT) cells. **(A)** A375 cells were exposed to a range of concentrations (0–500 μM) of **(I)** *rac*-SK-1, **(II)** *rac*-SK-2, **(III)** *rac*-SK-3, **(IV)** *L*-SK-4, **(V)** *D*-SK-4 and **(VI)** *rac*-SK-5 for 24, 48 and 72 h; **(B)** HaCaT and **(C)** A431 cells were exposed to a range of concentrations (0–500 μM) of *L*-SK-4 for 24, 48 and 72 h. Data shown are means ± SD of 3 replicates from three independent experiments. Asterisk (*), hashtag (^#^) or rhombus (^◊^) denote statistical significance when compare to their respective control at *p* < 0.05. **, ^##^ and ^◊◊^ denote statistical significance at *p* < 0.01 whereas ^***^, ^###^, ^◊◊◊^ at *p* < 0.001

A kinetic characterization of the ability of HOPO-based metal chelators [1,2-HOPO (*rac*-SK-3), 2,3-HOPO (*rac*-SK-5), 3,4-HOPO (*rac*-SK-2, *D*/*L*-SK-4) and hydroxypyranone (*rac*-SK-1)] to induce ROS generation was evaluated in malignant melanoma cells. Each compound was assessed at a range of concentrations (25–500 μM) and over 3 time points (24, 48 and 72 h). Our results show that none of the *rac*-SK-1, *rac*-SK-2, *rac*-SK-3 and *rac*-SK-5 compounds were able to induce ROS generation (Figs. 1AI-III and VI). On the other hand, two enantiomerically pure forms of an *N*-substituted-3, 4-HOPO had the capacity to induce a significant elevation of ROS at a concentration of 100 μM after 24 h of exposure (Figs. 1AIV and V). In particular, when A375 cells were treated with 100 μM of *L*-SK-4, substantially increased levels of ROS were maintained over the course of 72 h; whereas, in the case of *D*-SK-4 there was only a slight and dose-dependent elevation of ROS levels throughout the time of exposure (Figs. 1AIV and V). Additionally, the decrease in ROS induced-levels at higher concentrations (500 μM) suggested that *L*-SK-4 has completely chelated the redox active metals (e.g., Fe(III) and/or Cu(II)) that could be present in different cellular organelles, therefore minimizing the ROS production levels. These observations demonstrated the dual character of *L*-SK-4, which at 100 μM can act as a pro-oxidant, whereas at higher concentrations (500 μM) acts as an anti-oxidant. Finally, under similar experimental conditions, we evaluated the capacity of *L*-SK-4 to induce ROS generation against A431 as well as non-tumorigenic HaCaT cells in an attempt to document the selectivity of *L*-SK-4 against A375 cells. Our observations revealed that ROS levels also affected both A431 and HaCaT cells upon treatment with *L*-SK-4 but at a significantly lower magnitude (Fig. 1B and C). Taken together, our data indicate that *L*-SK-4 exerts a higher degree of potency in inducing elevated ROS generation in A375 compared to A431 and HaCaT cells.

### Effect of oxidative stress in lipids, proteins and DNA

**Fig. 2:**
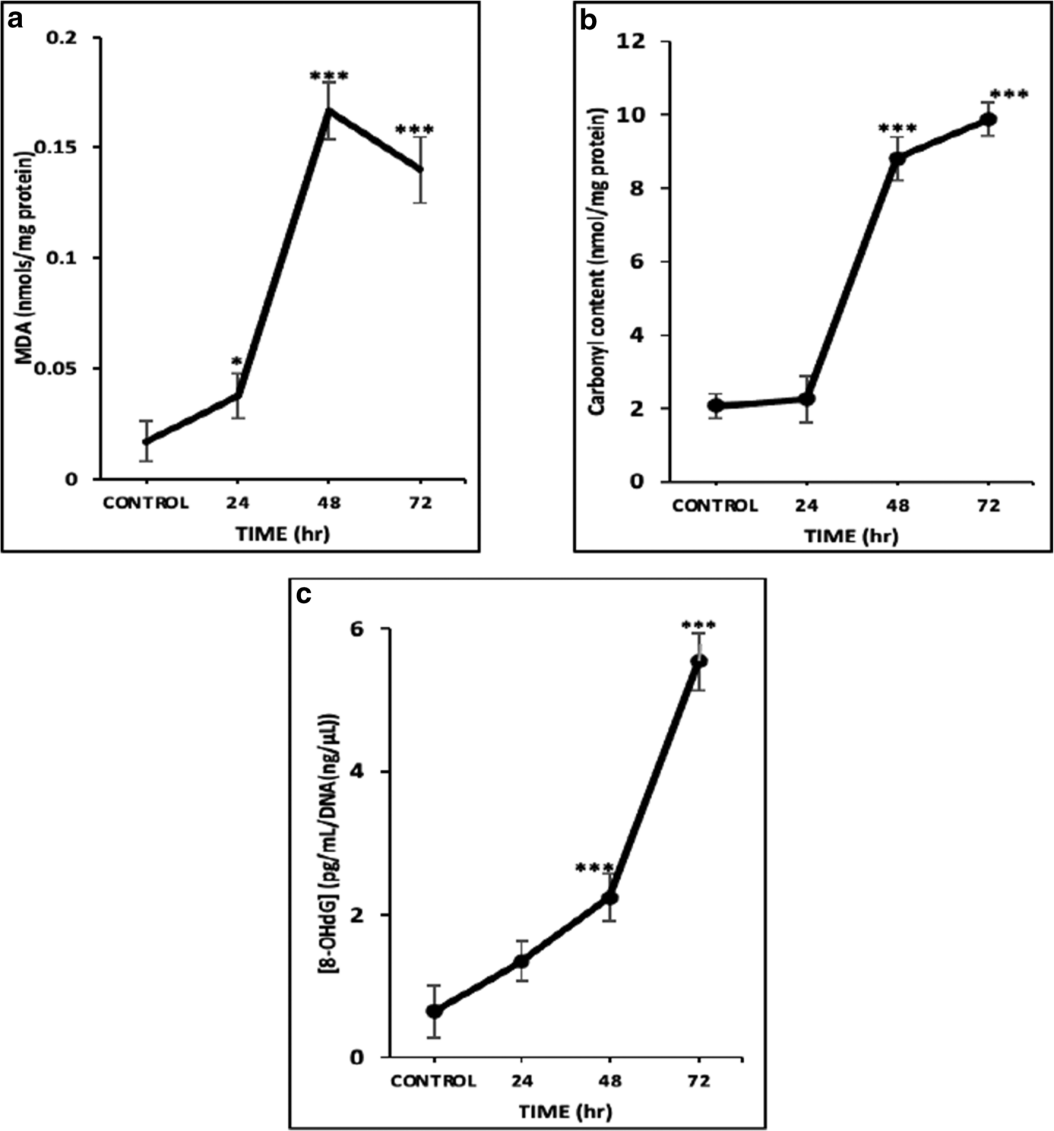
The effect of *L*-SK-4-induced ROS generation on lipids, proteins and DNA in melanoma cells. **(A)** Malondialdehyde (MDA), **(B)** protein carbonyl and **(C)** oxidative DNA damage (8-OHdG) contents upon treatment of A375 cells with 100 μM of *L*-SK-4 for 24, 48 and 72 h. Data shown are means of ± SD of 3 replicates from three independent experiments. Asterisk (*), denotes statistical significance when compare to their respective control at *p* < 0.05. ** denotes statistical significance at *p* < 0.01 whereas ^***^at *p* < 0.001

Then, we sought to determine the nature of ROS generation by means of assessing the content of lipid, protein and DNA oxidation levels. To this end, we firstly examined the levels of malondialdehyde content (MDA; a marker of lipid peroxidation) which were significantly increased as early as 24 h (Fig. 2A). In addition, the levels of protein carbonyl (a marker of protein oxidation; Fig. 2B) and 8-OHdG (a marker of oxidative DNA damage; Fig. 2C) content showed a similar pattern of increase but with statistical significance noted as early as 48 h and throughout the entire time course respectively.

### ROS inhibition by GSH

**Fig. 3:**
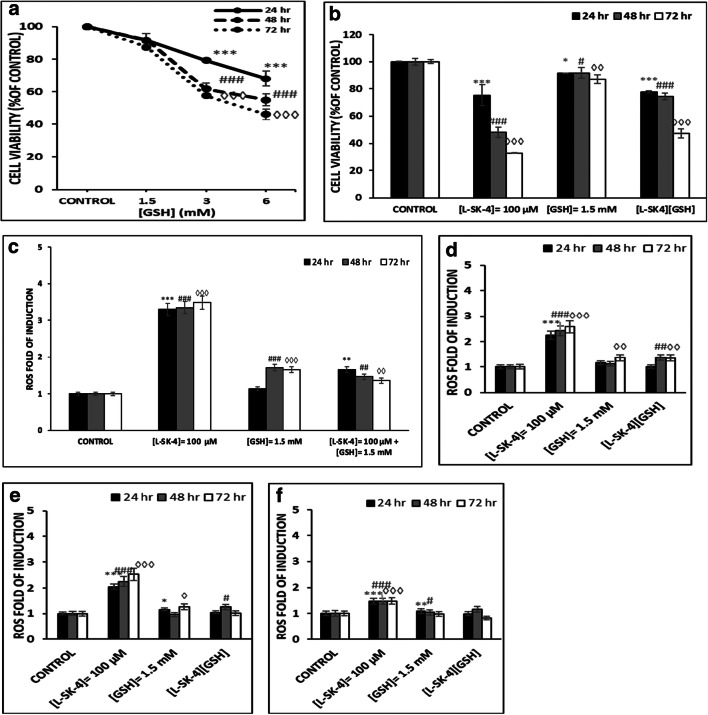
The effect of ROS quenching, by GSH, in melanoma cells. **(A)** A375 cells were exposed to a range of GSH concentrations (1.5–3.0 mM) for 24, 48 and 72 h; **(B)** A375 cells were treated with either 100 μM of *L*-SK-4 or pre-treated with 1.5 mM of GSH, for 2 h, and then co-treated with 1.5 mM of GSH and 100 μM of *L*-SK-4 for 24, 48 and 72 h; **(C)** GSH scavenges *L*-SK-4-induced ROS generation in melanoma cell lines; A375 cells were treated with either 100 μM of *L*-SK-4 or pre-treated with 1.5 mM GSH, for 2 h, and then co-treated with 1.5 mM of GSH and 100 μM of *L*-SK-4 for 24, 48 and 72 h; ROS levels were analyzed by flow cytometry and quantitated as ROS fold induction. Same experimental conditions were followed for (**D)** VMM1; **(E)** Hs294T and **(F)** B16F-10 melanoma cells. Data shown are means ± SD of 3–5 replicates from three independent experiments. Asterisk (*), hashtag (^#^) or rhombus (^◊^) denote statistical significance when compare to their respective control at p < 0.05. **, ^##^ and ^◊◊^ denote statistical significance at p < 0.01 whereas ^***^, ^###^, ^◊◊◊^ at p < 0.001

In subsequent experiments, we utilized glutathione (GSH; a well-known ROS scavenger [19, 20]) in order to examine the role of ROS inhibition under the same experimental conditions. A dose-response curve was obtained, in A375 cells, over a range of GSH concentrations (1.5–6.0 mM) at different time points (24–72 h) for determining the optimum experimental conditions of its usage (Fig. 3A). Our data indicated that a GSH concentration of 1.5 mM was well tolerated by the cells as at higher concentrations (3 and 6 mM) a cytotoxic effect was observed (Fig. 3A). Co-treatment of 1.5 mM GSH with 100 μM of *L*-SK-4 almost abolished the cytotoxic effect of the drug (Fig. 3B) due to inhibition of ROS production (Fig. 3B and C). In addition, we examined the degree of potency of *L*-SK-4 treatment in other melanoma cell lines including brain (VMM1; Fig. 3D) and lymph node metastatic melanoma (Hs294T; Fig. 3E) as well as murine non-metastatic melanoma (B16F-10; Fig. 3F) cells. From our results, it was evident that treatment with *L*-SK-4 induced a similar trend in increasing ROS levels, during the first 24 h, which was sustained at each time point thereafter. Finally, co-treatment with GSH and *L*-SK-4 also showed a significant reduction in ROS levels in a manner, similar to that observed in A375 cells (Figs. 3D-F).

### GSH prevents L-SK-4-induced apoptosis by ROS

**Fig. 4:**
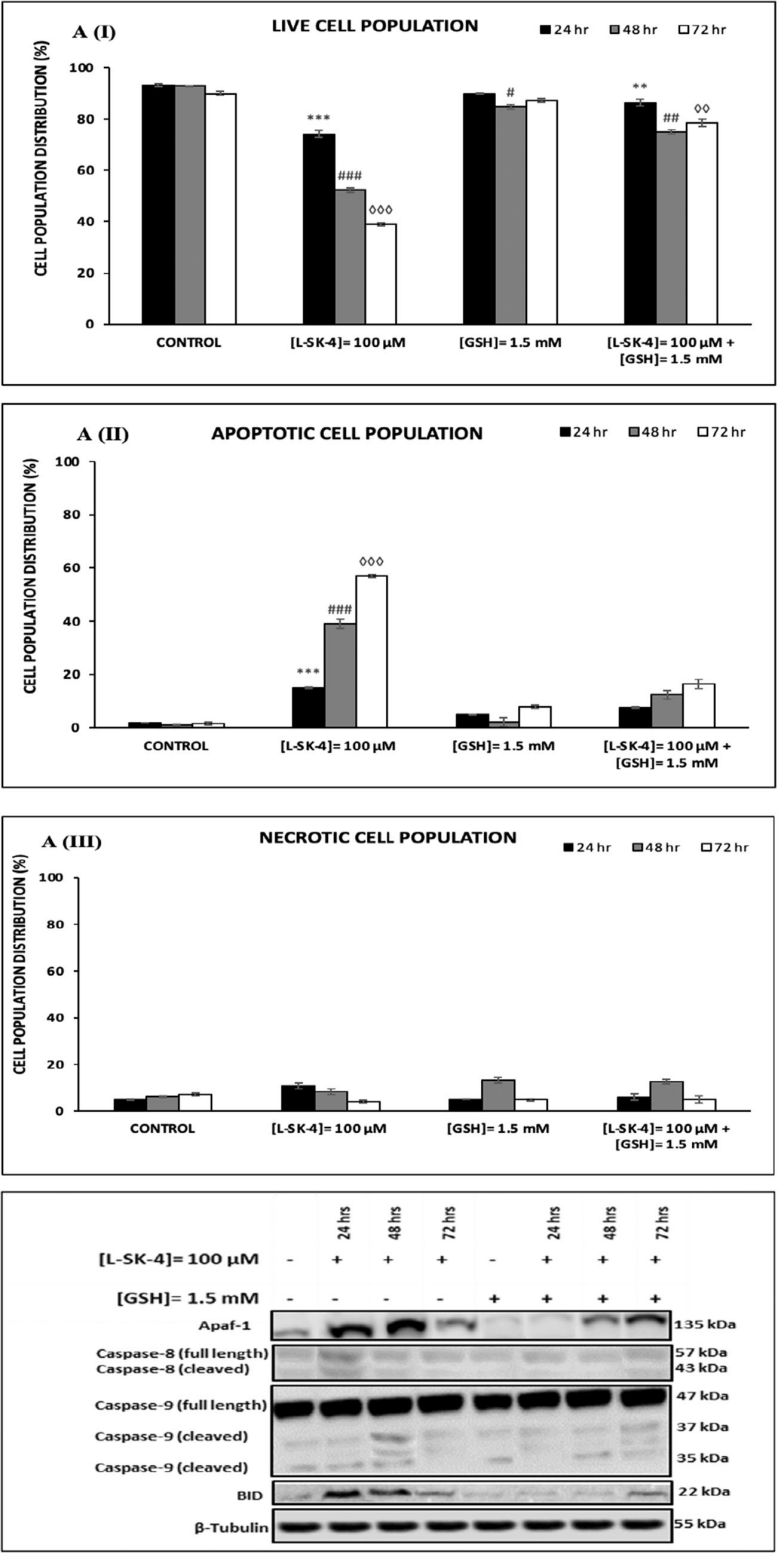
GSH prevents *L*-SK-4-induced apoptosis in melanoma cells. **(A)** A375 cells were either treated with 100 μM of *L*-SK-4 or pre-treated with 1.5 mM of GSH, for 2 h, and then co-treated with 1.5 mM of GSH and 100 μM of *L*-SK-4 for 24, 48 and 72 h. A flow cytometry-based approach was utilized for identifying and quantitating percent of **(I)** viable, **(II)** apoptotic and **(III)** necrotic cell populations; **(B)** Expression levels of full length and cleaved Caspases-8 and -9, BID as well as Apaf-1 proteins were recorded by western immunoblotting

Moreover, we determined the levels of apoptotic and necrotic cell populations in A375, VMM1, Hs294T and B16F-10 cell lines after exposure to *L*-SK-4, in the presence or absence of GSH, by utilizing a flow cytometry-based approach. Overall, our data showed a reduction in the levels of viable cells together with increased rates of apoptosis while levels of necrotic cells remained relatively low (Figs. 4AI-III). Additionally, it appeared that treatment with *L*-SK-4 was cytotoxic in the other melanoma cell lines as well including those of VMM1 (Online Resource 1; Fig. S1), Hs294T (Online Resource 2; Fig. S2) and B16F-10 (Online Resource 3; Fig. S3); although they were more resistant compared to A375 cells. Furthermore, necrotic induction rather than apoptotic was recorded in VMM1 (Online Resource 1; Fig. S1), Hs294T (Online Resource 2; Fig. S2) and B16F-10 (Online Resource 3; Fig. S3) cells. However, co-treatment with GSH had a beneficial effect as it reduced the levels of both apoptotic and necrotic cell populations. Finally, we aimed to identify key apoptotic proteins capable of modulating the intrinsic and extrinsic apoptotic cascades in response to *L*-SK-4 treatment (Fig. 4B). To this end, we evaluated the expression levels of i) cleaved and full length Caspase-8 (indicative of extrinsic apoptosis), ii) cleaved and full length Caspase-9 (indicative of intrinsic apoptosis), iii) apoptotic protease-activating factor-1 (Apaf-1; indicative of intrinsic apoptosis) and iv) BID (linker of extrinsic and intrinsic apoptotic cascades). Overall, our results indicated the activation of Caspase-8, BID, Caspase-9 and Apaf-1 (Fig. 4B) all of which have been previously reported to eventually lead to activation of Caspase-3 and consequently the execution of apoptosis [21–24]. In contrast, when cells were co-treated with GSH, the levels of the above-mentioned proteins were maintained at control levels, suggesting that inhibition of ROS generation (and consequently accumulation) is strongly associated with the activation of apoptosis (Fig. 4B). However, at 48 h post co-treatment, the levels of the listed proteins were increased; nonetheless, at a lesser extent compared to the *L*-SK-4 treated groups.

### Lipidomic profile of the L-SK-4 treated cells

**Fig. 5:**
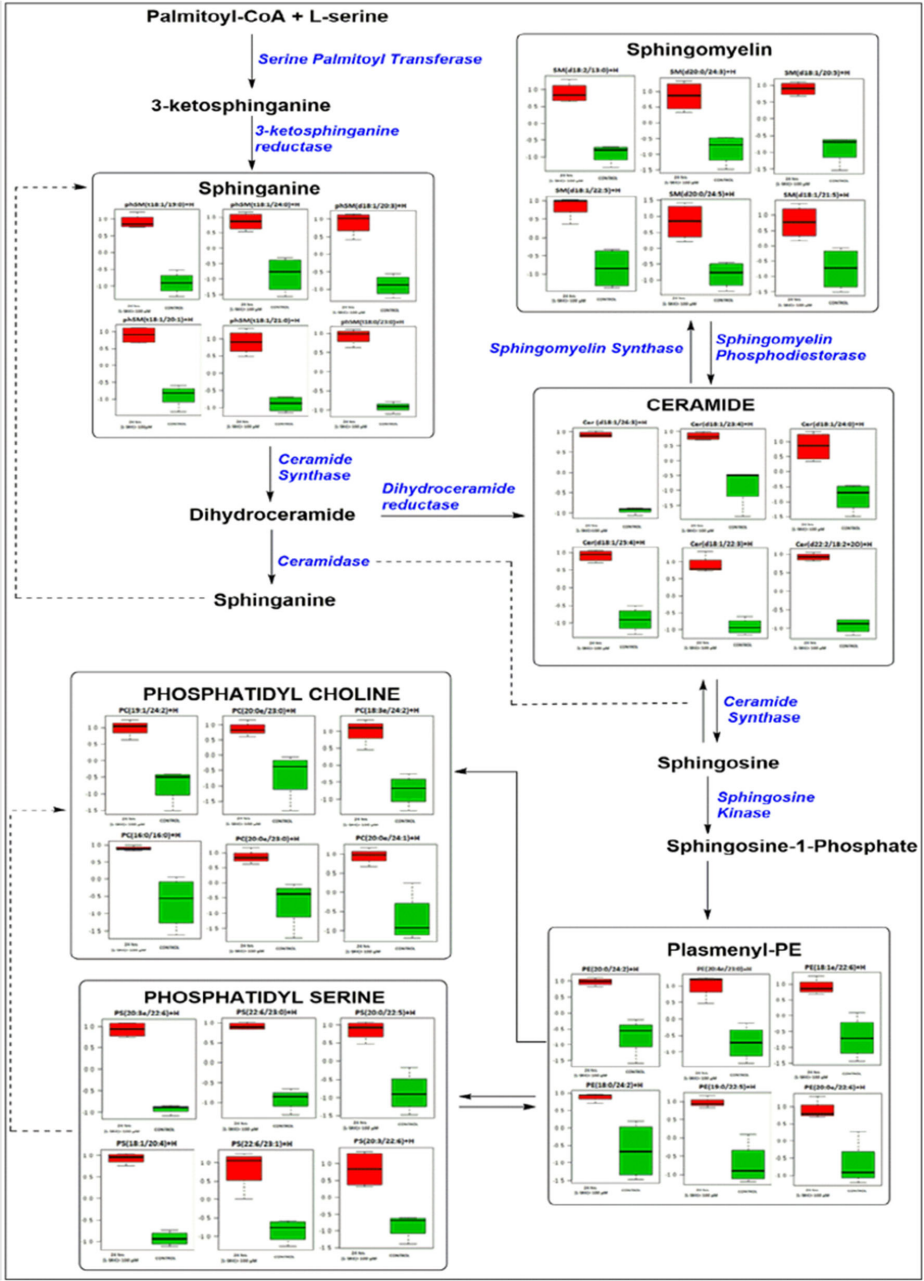
*L*-SK-4-induced sphingolipids biosynthesis in melanoma cells. A lipidomic analysis-based sphingolipid de novo biosynthetic map shows an up-regulation of sphingolipids biosynthesis after treatment with 100 μM of *L*-SK-4 (red bars) compared to the respective (untreated) controls (green bars)

In order to investigate further into how ROS generation modulates the apoptotic response, we performed a lipidomic profiling analysis, in A375 cells, after a 24 h treatment with 100 μM *L*-SK-4. PCA analysis and visualization indicated a planar separation between control, treated and quality control samples. It was revealed that 1800 lipid MS features (detected by MS1, *n* = 4) have been significantly dysregulated between control and treatment groups (Online Resource 4; Fig. S4). Partial least square-discriminate analysis (PLS-DA, 1 latent variable) was then applied to identify the most statistically significant features (VIP x > 1), recognizing 486 lapidated features that were retained (Online Resource 5; Fig. S5). Receiver operating curve (ROC) analysis using Monte-Carlo cross validation was then applied to elucidate the top 40 most significant features. From those, it was confirmed (by matching the MS1; m/z and processing the features via the bioinformatic platform; Thermo Scientific Lipid Search v4) that the elevated lipid classes were associated with de novo biosynthesis of sphingolipids with ceramides being the most significant ones (Fig. 5 and Online Resource 6; Fig. S6).

### The role of sphingolipids in ROS-induced apoptosis

**Fig. 6:**
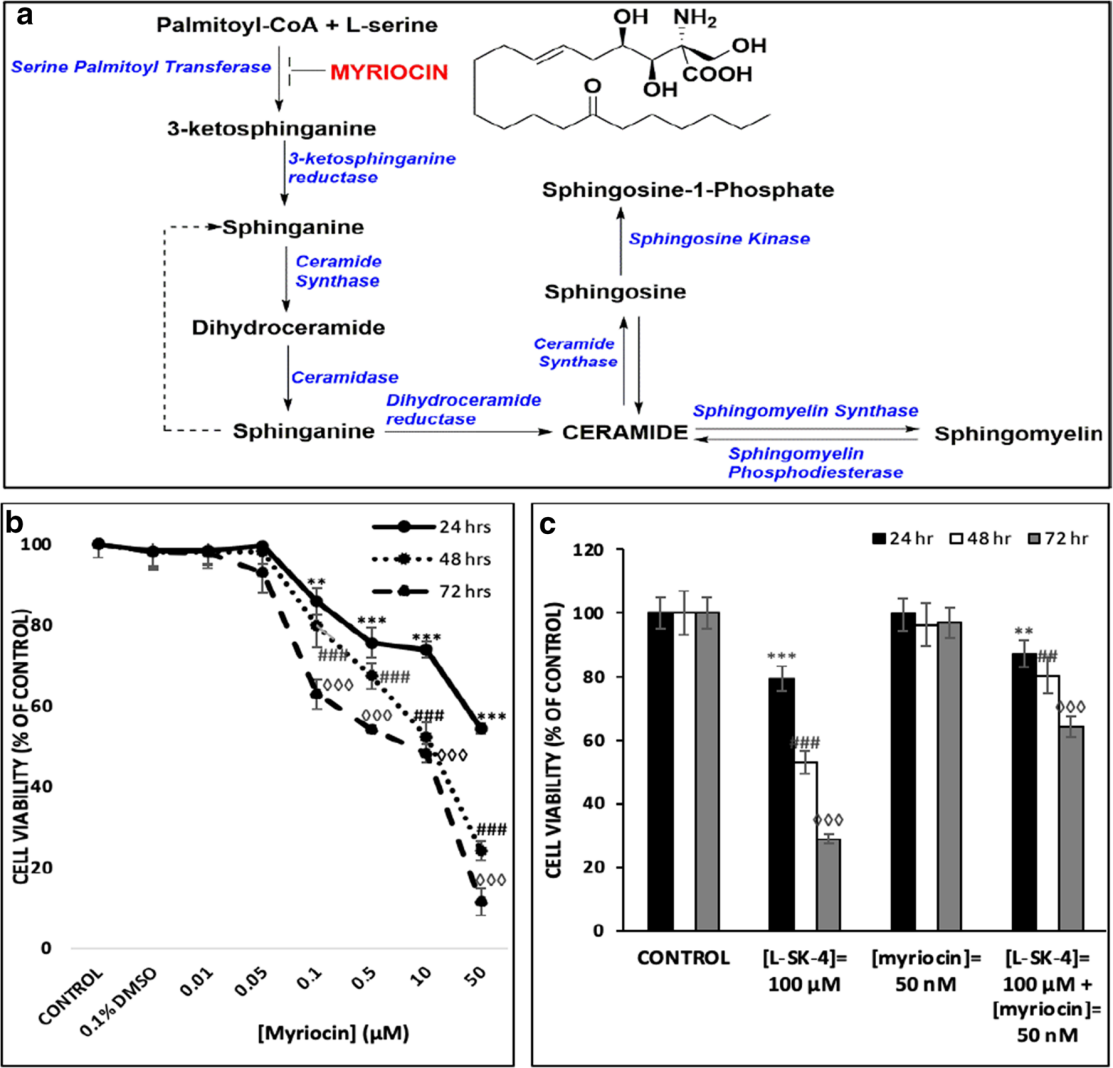
The effect of Myriocin on *L*-SK-4-induced cytotoxicity in melanoma cells. **(A)** Schematic presentation of Myriocin preventing ceramide biosynthesis by inhibition of Serine Palmitoyl Transferase; **(B)** A375 cells were treated with a range (0.01–50 μM) of Myriocin concentrations for 24, 48 and 72 h; **(C)** A375 cells were treated with either 100 μM of *L*-SK-4 or pre-treated with 50 nM of Myriocin, for 4 h, and then co-treated with 50 nM of Myriocin and 100 μM of *L*-SK-4 for 24, 48 and 72 h. Data shown are means ± SD of 5 replicates from three independent experiments. Asterisk (*), hashtag (^#^) or rhombus (^◊^) denote statistical significance when compare to their respective control at p < 0.05. **, ^##^ and ^◊◊^ denote statistical significance at p < 0.01 whereas ^***^, ^###^, ^◊◊◊^ at p < 0.001

According to the literature, elevation in ROS generation can stimulate the release of ceramide species which can act as second messengers for activating apoptosis [25]. In order to validate if a ROS-driven elevation of ceramide levels could induce apoptosis, we utilized Myriocin (an inhibitor of serine palmitoyl transferase [26, 27]) as a treatment vehicle to inhibit de novo biosynthesis of sphingolipids (Fig. 6A). Initially, A375 cells were exposed to a range of Myriocin concentrations (0–50 μM) over various time periods (24–72 h) for determining the optimum experimental conditions of its usage (Fig. 6B). As a result, A375 cells were treated with 100 μM of *L-*SK-4 in the presence or absence of 50 nM Myriocin. As expected, the viability levels of A375 cells co-treated with *L*-SK-4 and Myriocin were significantly elevated implying that the utilization of Myriocin partly rescued cells from the observed *L*-SK-4-induced cytotoxicity (Fig. 6C).

**Fig. 7:**
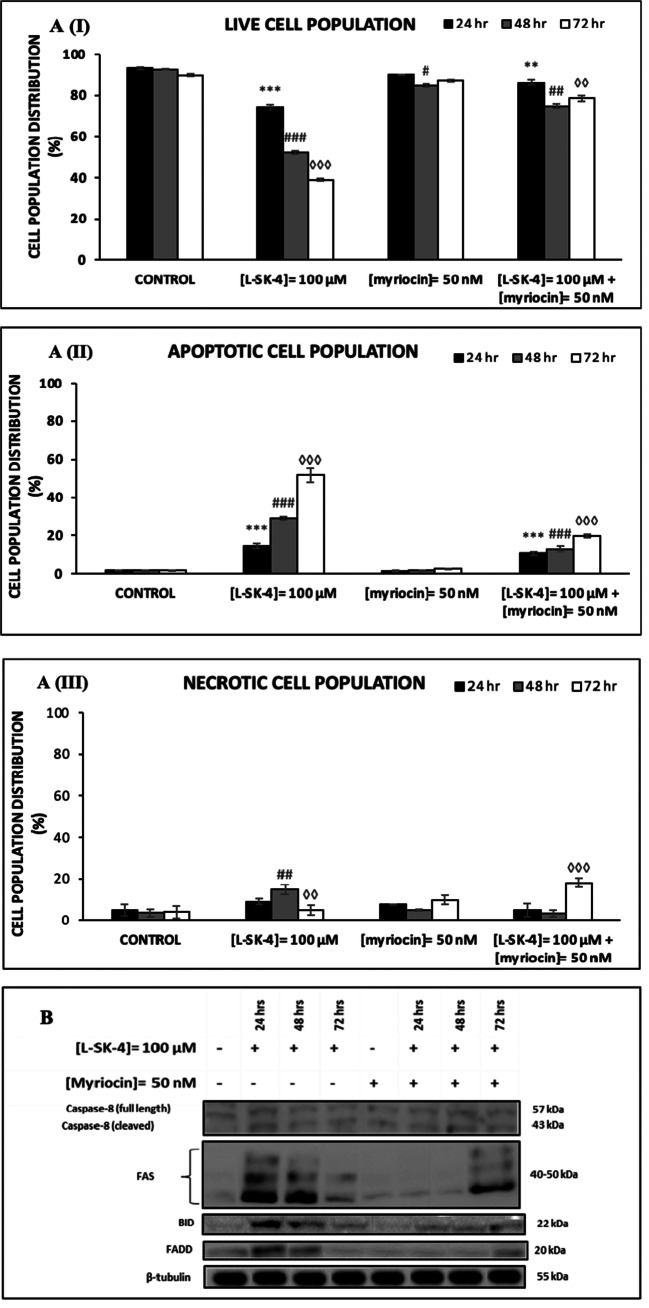
Myriocin prevents *L*-SK-4-induced extrinsic apoptosis in melanoma cells. **(A)** A375 cells were treated with 100 μM of *L*-SK-4 or pre-treated with 50 nM of Myriocin, for 4 h, and then co-treated with 50 nM of Myriocin and 100 μM of *L*-SK-4 for 24, 48 and 72 h. A flow cytometry-based approach was utilized for identifying and quantitating percent of **(I)** viable, **(II)** apoptotic and **(III)** necrotic cell populations; **(B)** Expression levels of full length and cleaved Caspase-8 as well as BID, FADD and FAS proteins were recorded by western immunoblotting

Next, we sought to evaluate the effect of Myriocin on *L*-SK-4-induced cell death by means of flow cytometry (Fig. 7AI-III). A similar pattern was observed, in A375 cells co-treated with *L*-SK-4 and Myriocin, by means of increased levels of viable (Fig. 7AI) and decreased levels of apoptotic cells (Fig. 7AII) while those of necrotic remained relatively unaffected (Fig. 7AIII). The capacity of ceramide to activate extrinsic apoptosis (by inducing FasL stimulation) has been well documented in the literature [28, 29]. Therefore, we aimed to examine the underlying mechanism(s) triggering extrinsic apoptosis as a response to ceramide induction. For this reason, we determined the protein expression levels of Fas and Fas-associated death domain (FADD) as well as those of Caspase-8 and BID, in A375 cells. According to our data, treatment with *L*-SK-4 increased expression levels of Fas, FADD, Caspase-8 and BID, an effect which was significantly minimized after co-treatment with Myriocin; thus suggesting a role of ceramide in modulating the activation of extrinsic apoptosis in A375 cells (Fig. 7B.

**Fig. 8:**
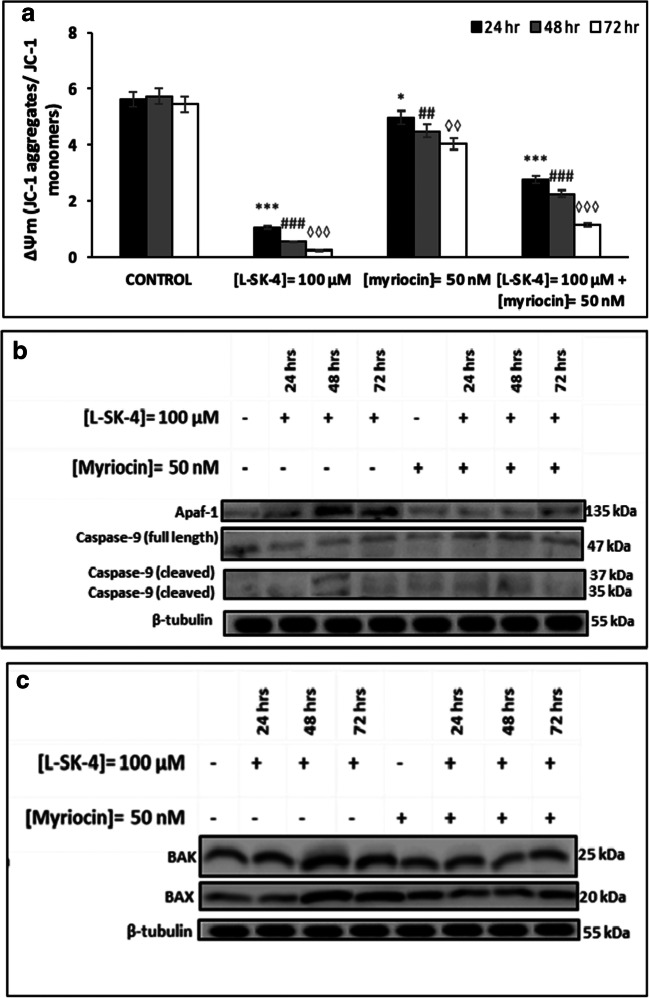
Myriocin prevents *L*-SK-4-induced intrinsic apoptosis in melanoma cells. **(A)** A375 cells were treated with either 100 μM *L*-SK-4 or pre-treated with 50 nM of Myriocin, for 4 h, and then co-treated with 50 nM of Myriocin and 100 μM of *L*-SK-4 for 24, 48 and 72 h. A flow cytometry-based approach was utilized for quantitating mitochondrial membrane depolarization as the ratio of JC-1 aggregates over JC-1 monomers. Expression levels of **(B)** full length and cleaved Caspase-9 as well as Apaf-1 proteins in addition to **(C)** pro-apoptotic proteins BAK and BAX were recorded by western immunoblotting

Finally, we attempted to further investigate into the effect of increased sphingolipid biosynthesis on the activation of intrinsic apoptosis. For this reason, we utilized flow cytometry and western immunoblotting-based approaches in order to examine levels of mitochondrial membrane depolarization (ΔΨ_m_) (by utilizing JC-1 as a marker of mitochondrial dysfunction; [30, 31]) (Fig. 8A) as well as those of Apaf-1, Caspase 9 and pro-apoptotic regulators BAX and BAK respectively (Fig. 8B and C). According to our results, treatment with 100 μM of *L*-SK-4 induced a dramatic depolarization of mitochondria while co-treatment with Myriocin re-polarized the mitochondrial membrane, to a variable degree, in A375 cells (Fig. 8A). Furthermore, we examined the expression levels of those proteins closely associated with the formation of apoptosome; namely, Apaf-1 and Caspase-9 both of which were shown to be activated under *L*-SK-4 treatment in A375 cells. In contrast, co-treatment with Myriocin caused a significant reduction in the expression of these proteins (Fig. 8B). In addition, we examined the expression levels of pro-apoptotic proteins BAX and BAK as markers of mitochondria degradation [32, 33] (Fig. 8C). Our data indicated their activation under treatment with *L*-SK-4, an effect which was reversed in the presence of Myriocin, suggesting that ceramide can also induce the activation of pro-apoptotic proteins; thereby, leading to the activation of downstream components of the intrinsic apoptotic cascade.

## Discussion

Overexpression of anti-oxidant enzymes leads to an enriched anti-oxidant capacity in human malignant melanoma cells thus allowing them to quench excessive generation of ROS [34, 35]. Previous studies comparing the anti-oxidant capacity between human melanoma cells and normal melanocytes have revealed that melanoma cells possess significantly lower concentrations of intracellular GSH, ferritin, ubiquinone and catalase therefore making them more vulnerable to extracellular peroxides [36–38]. In contrast, elevated levels of superoxide dismutase (SOD) and vitamin E makes them resistant to oxidative stress while overexpression of NADPH oxidase 1 (induced by intracellular ROS) and superoxide anion are involved in malignant transformation and invasiveness of melanoma [39–41]. Consequently, it can be concluded that the anti-oxidant capacity of melanoma cells is pivotal for their growth and proliferation [42]. On the other hand, other studies have suggested that induction of oxidative stress is responsible for making the anti-oxidant enzymes unable to cope with the toxic levels of ROS, thus leading to excessive ROS accumulation, lipid peroxidation, protein carbonylation and oxidative DNA damage all of which can trigger apoptotic cell death and therefore can be the target of new therapeutic strategies in malignant melanoma [39].

The capacity of metal chelators to exert an anti-cancer activity has been reported in a variety of in vitro cancer models [43–45]. In a previous study published by our group, we have documented the anti-cancer ability of a newly synthesized series of hydroxypyridinone-based analogues of *L*-Mimosine in an in vitro model of malignant melanoma. From these compounds, a methylated *N*-substituted hydroxypyridinone form was shown to be the most cytotoxic by exerting a higher potency against A375 than A431 and HaCaT cells [18]. Herein, we performed a kinetic determination of ROS induction in a series of HOPO-based analogues of *L*-Mimosine in an attempt to characterize the mode of action of this class of compounds. Our data suggest that both enantiomers (*L-* and *D-*) of the compound have the ability to induce ROS generation with the *L-* one being more potent (4X) compared to untreated control as well as showing a selectivity for A375 rather than HaCaT and A431 cells.

Co-treatment of GSH and *L-*SK-4 significantly rescued A375, VMM-1, Hs294T and B16F-10 cells from apoptosis, by reducing ROS levels. This can be attributed to the fact that melanoma cells have generally low levels of GSH and thus are not able to overcome the induction of high levels of ROS generation caused by *L*-SK-4 [43]. Moreover, although induction of both necrosis and apoptosis were recorded, the magnitude of such potency was different among different melanoma cell lines in a way where Hs 294 T, VMM-1 and B16F10 ones were more resistant compared to the A375 one. These findings were validated by western immunoblotting revealing the activation of key apoptotic proteins involved in both intrinsic and extrinsic apoptotic cascades. On the other hand, co-treatment with GSH reversed this trend and consequently rescued cells from apoptotic cell death indicating a key role of ROS in modulating the apoptotic response. Our findings are in agreement with other studies showing that metal chelators can induce ROS-driven apoptosis [43] and co-treatment with GSH can prevent apoptotic activation [46, 47].

In an attempt to look further into the underlying mechanisms of how *L-*SK-4-induced generation of oxidative stress modulates the apoptotic response, we employed a lipidomic approach in A375 cells. A significant up-regulation of sphingolipid levels was observed with emphasis on those of sphinganine, sphingomyeline, ceramide, phosphatidyl choline, phosphatidyl serine and plasmenyl phosphatidyl ethanolamine. These findings are in agreement with other studies indicating that ROS elevation stimulates the biosynthesis and release of ceramide lipids (as well as other sphingolipids) via ceramide releasing enzymes resulting in generation of a ceramide-enriched membrane [48–50]. Moreover, additional experiments with Myriocin (an inhibitor of the first step of ceramide biosynthesis) confirmed the validity of our observations as inhibition of ceramide synthesis led to the rescue of cells by preventing apoptotic activation. Such preventive action has been explored by other studies suggesting that ceramide (as a bioactive lipid molecule) can act as a second messenger capable of activating directly or indirectly the extrinsic and intrinsic apoptotic pathways respectively [51, 52].

To conclude, we have shown that an N-substituted-3.4-HOPO was the most potent metal chelator capable of inducing cytotoxicity in various melanoma cell lines. Its suggested mode of action involves increased ROS production resulting in significantly increased levels of sphingolipids’ (ceramide) biosynthesis which, in turn, can act as second messenger(s) capable of mediating the intrinsic as well as extrinsic apoptosis thereby modulating the therapeutic response of this newly synthesized *L*-SK-4 compound.

## Supplementary Information

ESM 1 (PDF 1.07 mb)

## Declarations

### Availability of data and material

All data generated or analysed during this study are included in this published article and its supplementary information files.
