# Supplementary material for: A novel methylated analogue of L-Mimosine exerts its therapeutic potency through ROS production and ceramide-induced apoptosis in malignant melanoma
Source: Invest New Drugs. 2021 Feb 23;39(4):971–86. doi: 10.1007/s10637-021-01087-5 (PMC8280034; doi:10.1007/s10637-021-01087-5)
Supplement: Supplementary file 1 — (PDF 1.07 mb) [file 10637_2021_1087_MOESM1_ESM.pdf]

SUPPLEMENTARY MATERIAL

A novel methylated analogue of *L*-Mimosine exerts its therapeutic potency through ROS production and ceramide-induced apoptosis in malignant melanoma

Sotiris Kyriakou[1,2,3], William Cheung[1], Theodora Mantso[1], Melina Mitsiogianni[1], Ioannis Anestopoulos[2,3], Stephany Veuger[1], Dimitris T. Trafalis[4], Rodrigo Franco[5,6], Aglaia Pappa[7], David Tetard[1] and Mihalis I. Panayiotidis[1,2,3*]

[1]Department of Applied Sciences, Northumbria University, Newcastle Upon Tyne, UK; [2]Department of Cancer Genetics, Therapeutics & Ultrastructural Pathology, The Cyprus Institute of Neurology & Genetics , Nicosia, Cyprus and [3]The Cyprus School of Molecular Medicine, The Cyprus Institute of Neurology & Genetics, Nicosia, Cyprus; [4]Department of Pharmacology, Medical School, National & Kapodistrian University of Athens, Athens, Greece; [5]Redox Biology Centre and [6]School of Veterinary Medicine & Biomedical Sciences, University of Nebraska, Lincoln, USA; [7]Department of Molecular Biology & Genetics, Democritus University of Thrace, Alexandroupolis, Greece

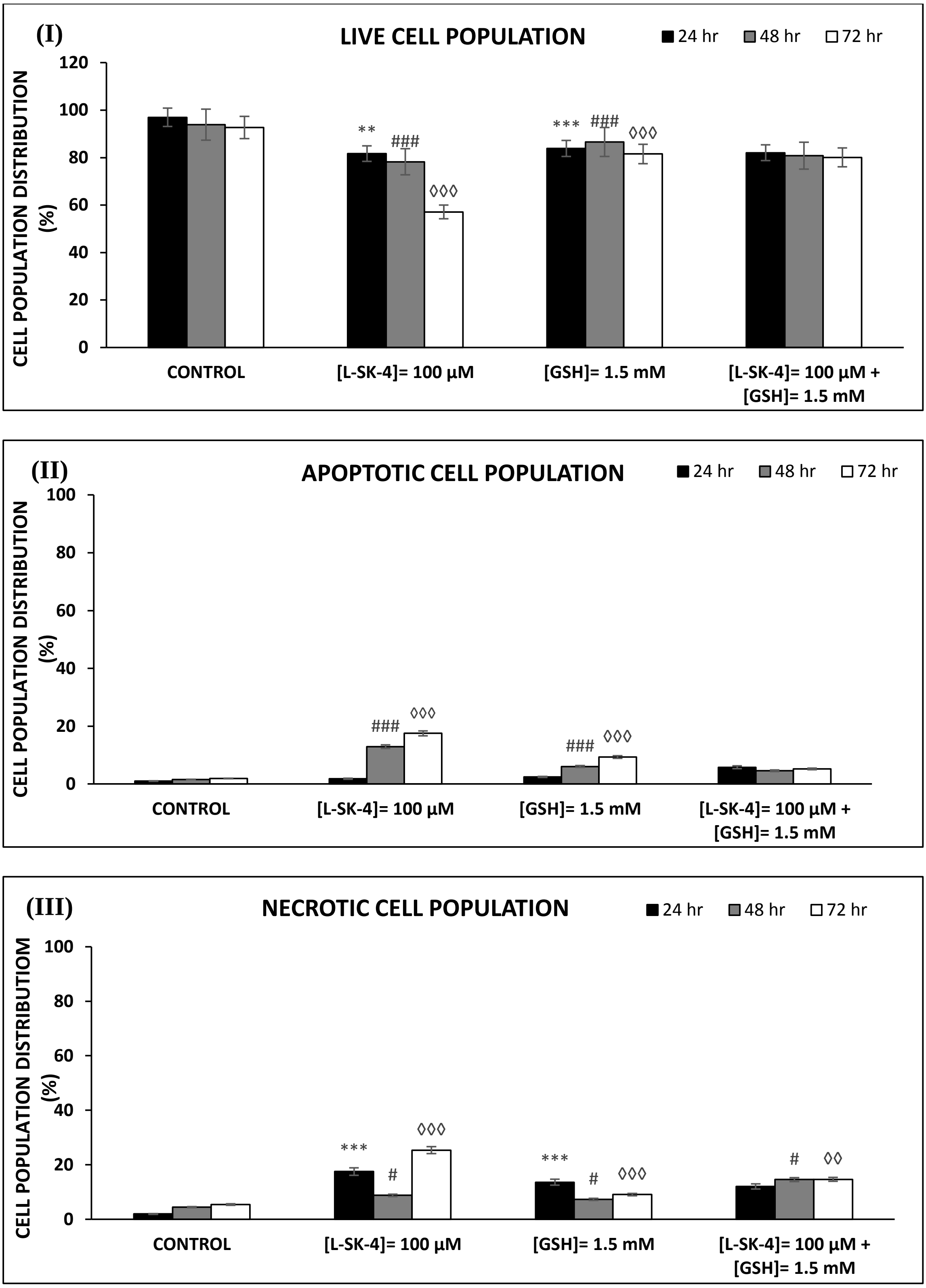

Figure S1: GSH prevents apoptotic induction in VMM-1 cells. Briefly cells were treated with 100 µM of *L*-SK-4 in the presence or absence of 1.5 mM of GSH for 24, 48 and 72 hrs. A flow cytometry-based approach was utilized for identifying live **(I)**, apoptotic **(II)** and necrotic **(III)** cell populations which were quantitated as percentages. Data shown are means of ± SD of 3 replicates from three independent experiments.

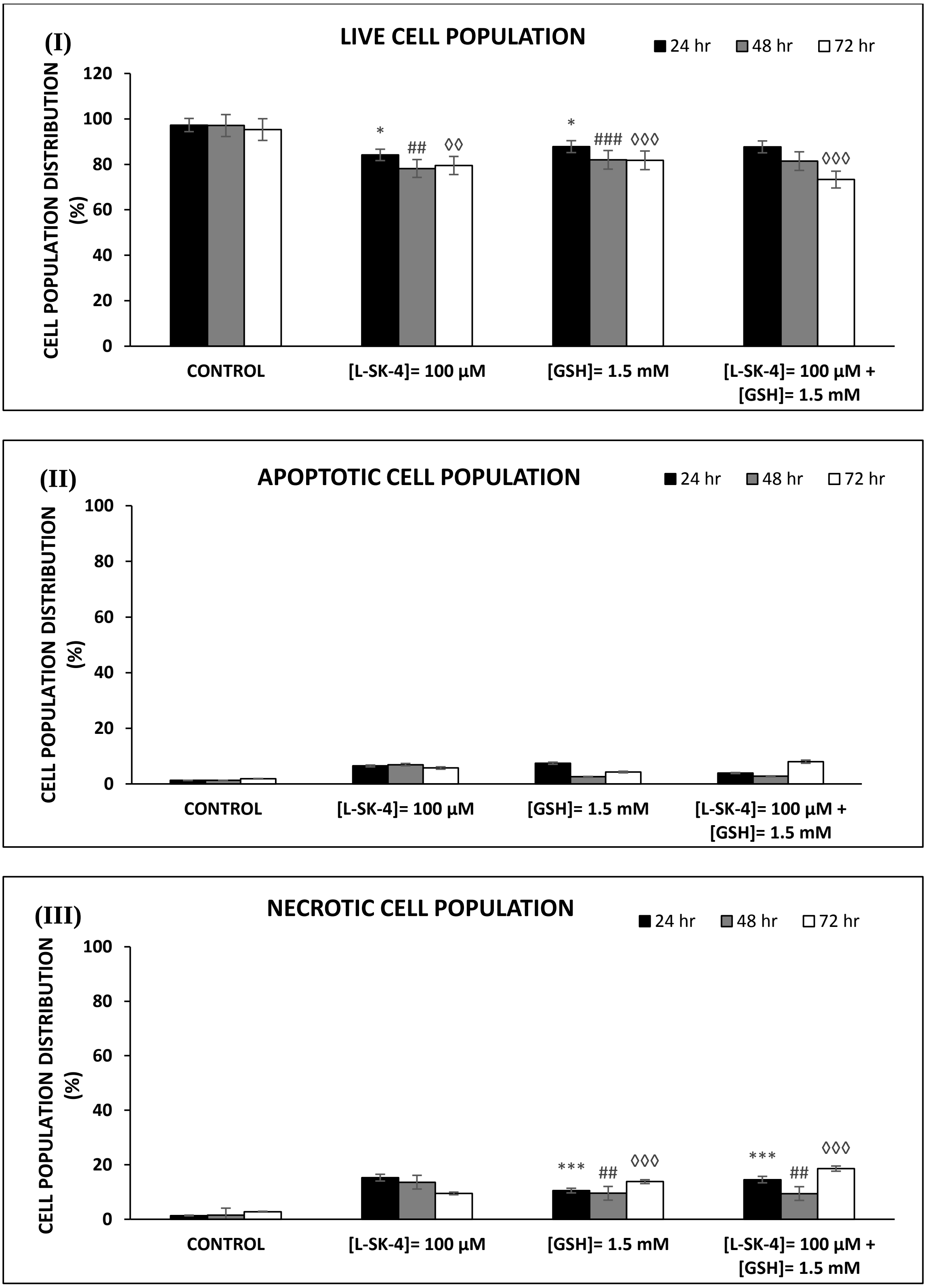

Figure S2. GSH prevents apoptotic induction in Hs 294T cells. Briefly cells were treated with 100 µM of *L*-SK-4 in the presence or absence of 1.5 mM of GSH for 24, 48 and 72 hrs. A flow cytometry-based approach was utilized for identifying live **(I)**, apoptotic **(II)** and necrotic **(III)** cell populations which were quantitated as percentages. Data shown are means of ± SD of 3 replicates from three independent experiments.

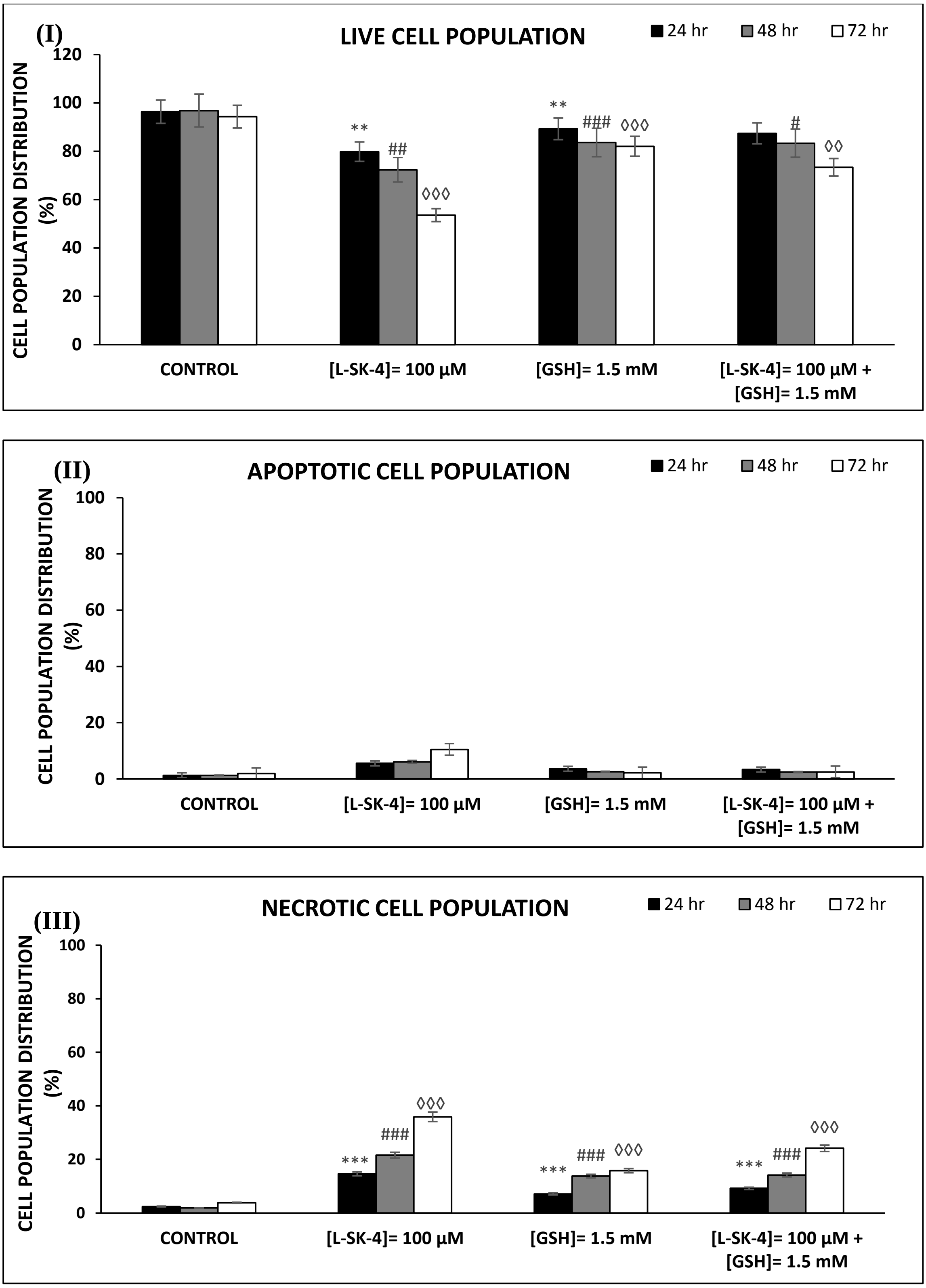

Figure S3. GSH prevents apoptotic and necrotic induction in B16F-10 cells. Briefly cells were treated with 100 µM of *L*-SK-4 in the presence or absence of 1.5 mM of GSH for 24, 48 and 72 hrs. A flow cytometry-based approach was utilized for identifying live **(I)**, apoptotic **(II)** and necrotic **(III)** cell populations which were quantitated as percentages. Data shown are means of ± SD of 3 replicates from three independent experiments.

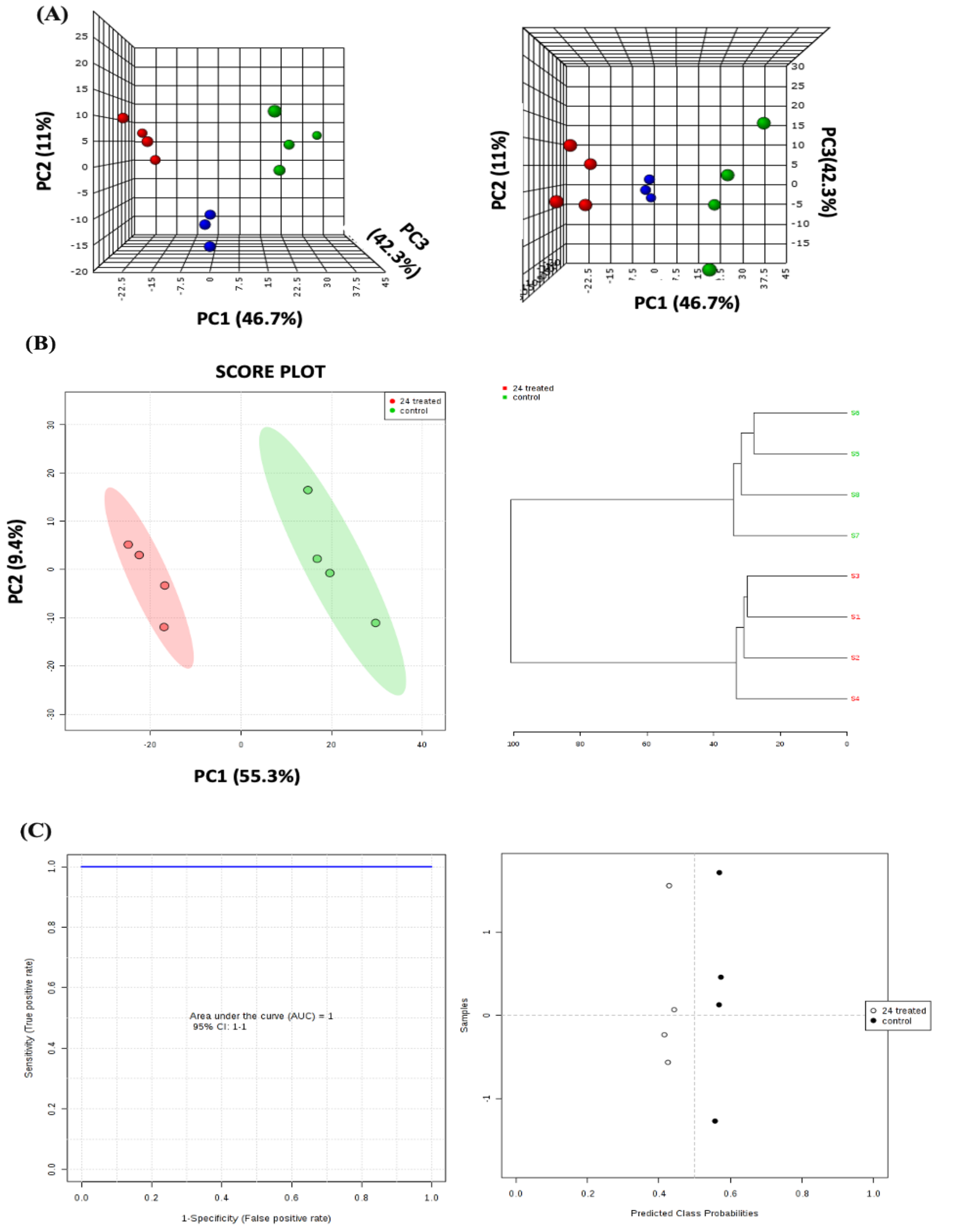

Figure S4: PCA visualization, raw data set approx. 1800 lipid MS features detected (MS1 profiling) n=4. Planar separation can be observed between treated (red spheres), control (green spheres) and quality control (blue spheres) (x~ 6% relative standard deviation (r.s.d) –analysis stability assessment. Extraction blank were also imbedded for background ion subtractions prior to PCA visualization. Control and treatment 95% confidence level are highlighted between each groups, approx. 1800 MS features**.**

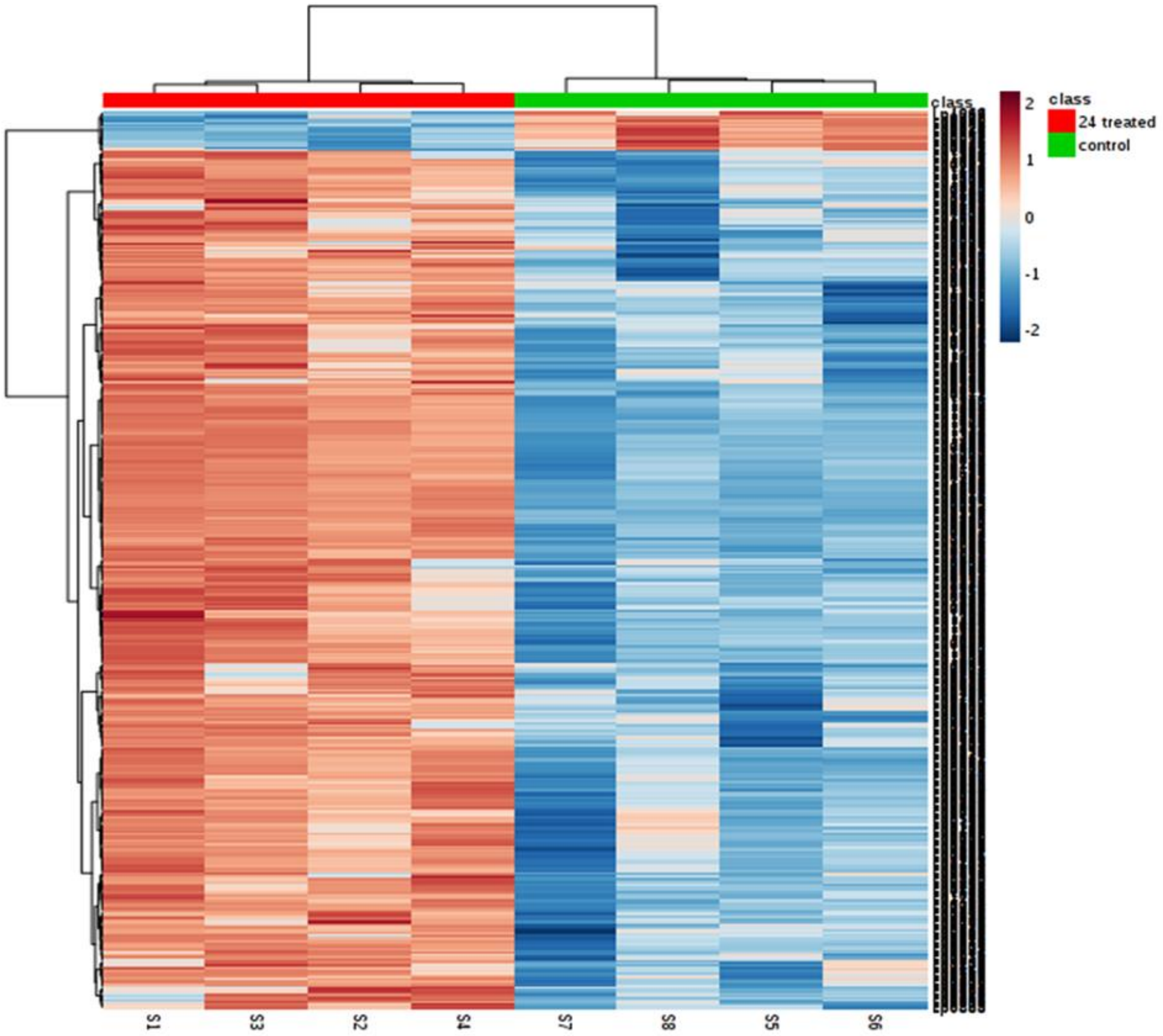

Figure S5: Heat map representation of the top 400 discriminate features, identified via ROC analysis, showing the dysregulation of the lipidome profile of A375 cells exposed to 100 µM *L*-SK-4, for 24 hrs, compared to the respective untreated control.

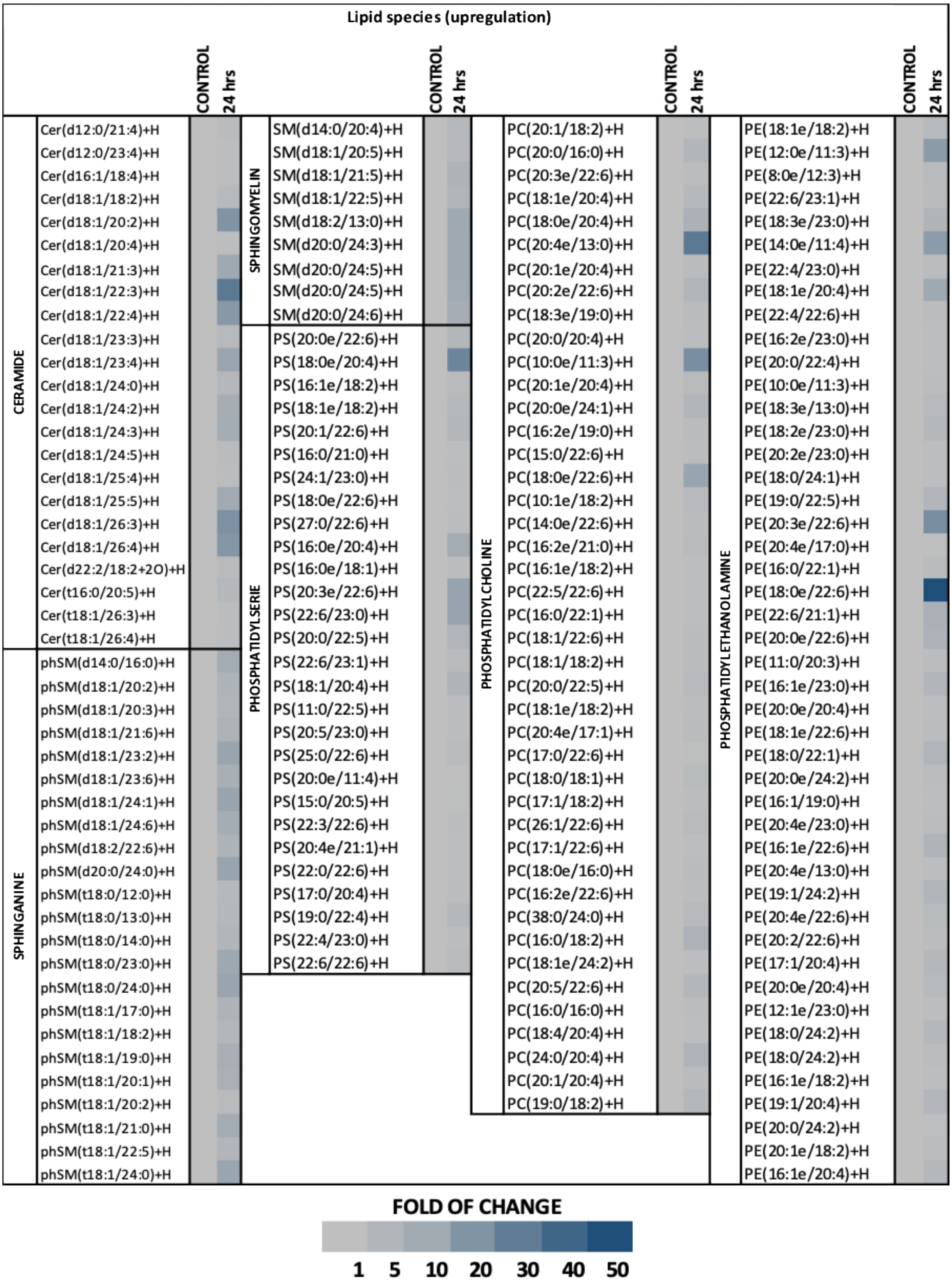

Figure S6: Heat map representation of the sphingolipids' profile of A375 cells between untreated and 24 hrs post treatment with 100 µM *L*-SK-4 experimental conditions.
